# Progress in Surgery of the Liver, Gall Ducts, Spleen, and Pancreas

**Published:** 1901-02-23

**Authors:** 


### Progress in Surgery of the Liver, Gall Ducts, Spleen, and Pancreas.

**Rupture of the Liver.**—If we are confronted with a case in which the diagnosis of rupture of the liver is made, and in which the immediate shock is rallied from—in other words, one in which the patient does not succumb in the first few hours to shock and hæmorrhage—we shall find that further developments will either point to the occurrence of peritonitis or to the slow or rapid accumulation of fluid in the abdominal cavity, with symptoms of loss of blood and a temperature which is peculiar. The peculiarity consists in a rise, with an unusual difference between the oral and rectal temperatures, which is pretty certainly caused by the process of absorption of degenerating and disintegrating blood clot. The patient's best chance is in operation. We must get rid of the blood clots and fluid blood, and control any hæmorrhage which may still be going on. Hot surgical salt solution will be found to be an exceedingly useful preparation in operations of this sort, both in the abdomen and for intravenous infusion.[1] Heaton[2] records a case of rupture of the liver and kidney successfully treated by abdominal section and nephrectomy.

**Hydatid Cysts of the Liver.**—Potherat[3] maintains that when in the same subject there exist either in the same organ, or in different viscera, or on the surface of the serous lining, multiple hydatid cysts, these multiple cysts are to be regarded as diffused manifestations of a single or repeated invasions of the embryos, and not as inoculations of the contents of the primary cyst. Other observers, however, think that there is strong clinical evidence that daughter vesicles from a ruptured hydatid cyst may become engrafted in other structures, and by subsequent growth may form secondary hydatid growths. Jalland[4] reports a case of large hydatid cyst successfully treated by laparotomy and drainage; and Clayton[5] records a case of hydatid disease of the liver which was discovered by the presence of daughter cysts in the motions. In the latter case the constant sequence of events—pain resembling biliary colic, followed by jaundice, which was always transient, the uniform size of the fragments passed, and the rupture of the larger cysts—strongly suggest: 1. That the cyst was discharging through the biliary passages. 2. That at times portions became infected, producing the colic. 3. That by spasm and irritation, or by rupture and discharge of the irritating contents of a cyst into these ducts, the jaundice was caused. Davis[6] reports the case of a hydatid cyst of about twenty-five ounces capacity situated between the liver and diaphragm. The case was successfully treated by operation.

**Disease of the Gall Tracts.**—Jones[7] lays stress on the following points:—1. The diagnostic value of the point of maximum tenderness on pressure, which is over the gall bladder at or near the costal margin of the ninth rib. This point in disease of the gall tracts corresponds in importance with McBurney's point in disease of the appendix. 2. The diagnostic value of the presence of bile in the urine excreted during or immediately after a very brief obstruction of the common duct. 3. Disease of the gall tracts is of very common occurrence, and is liable to be mistaken for other troubles which it closely imitates. In most cases it is wiser to do first a cholecystostomy, which is attended by the least risk, leaving more radical treatment for another time, should it become necessary. Dalziel[8] records seven cases of operations for gallstones in the bile ducts which were removed either through an incision in the duct wall or by opening the gall bladder and dilating the cystic duct. The incision through the abdominal wall, found most suitable as affording more room to work deep in the abdomen, is one in the form of an obtuse angle, one line passing from the tip of the tenth cartilage parallel to and one and a half inches from the costal margin towards the xiphoid cartilage, and the other perpendicular from the same point of origin, each limb of the angle being about three inches in length, and varying according to the thickness of the abdominal wall.

**Splenectomy.**—Bovec[9] reports a case of successful splenectomy for congestive hypertrophy, the organ weighing, in the fresh state, four pounds and four ounces. He places considerable emphasis upon the importance of observation of splenectomy cases, other than emergency operations, for a considerable amount of time previous to operation in order to be certain of the blood conditions, and to attempt to arrive at a conclusion as to the actual cause and character of the splenic condition. A blood count of 4,000,000 red and 6,000 to 9,000 white blood cells, and a hæmoglobin percentage above 70 per cent. would ordinarily be considered a safe blood condition for splenectomy. But should the red cells be only 2,500,000, and the white ones increased to above 9,000, and perhaps the hæmoglobin percentage be below 70, Bovec would conclude splenectomy under the most favourable conditions otherwise would be absolutely interdicted, unless death without operation seemed unavoidable. Splenectomy for malignant disease has not been satisfactory, as the disease spreads so rapidly. For echinococcus and other cysts the operation is very successful, as it has been in rupture of the organ. Splenopexy has been recommended for movable or wandering spleen when the organ is less than three times its normal size, but Bovec would apparently prefer splenectomy for this condition.

**Pancreatic Surgery.**—Several important contributions[10] have appeared on this subject. Ceccherelli[11] arranged his conclusions under twenty-four headings, of which the following are the principal:—1. Emaciation, presence of fat in the fæces, sugar in the urine, bronzed skin, jaundice, and pain are met with in the majority of pancreatic affections. 2. Surgical intervention is more justifiable at the small end of the pancreas than at the head. 3. Extirpation should not be attempted in cases of tuberculous or syphilitic lesions. In partial extirpation one of the two canals must be left. 4. Pancreatic tumours are generally either blood cysts following injuries or retention cysts. In the latter case surgical intervention is useful, but should be limited to excision of the cyst. It is necessarry to keep in mind the question of opening Wirsung's canal, and the probable discharge of pancreatic juice into the abdominal cavity. 5. Pancreatic calculi may be extracted. 6. Necrosed fragments of the pancreas may be removed. 7. In suppurative or gangrenous pancreatitis the rule is to do nothing during the acute stage. For the treatment of abscess or gangrene of the pancreas three routes are available—the lumbar extra-peritoneal, the transpleural, and the median supra-umbilical. 8. In hernia of the pancreas produced by injuries, reduction and even fixation may be performed. 9. In contusions and wounds of the pancreas, if there is hæmorrhage, it may be arrested either by suture or by tying the bleeding vessels. Clots of blood in the abdominal cavity should be removed at the same time. 10. Experimental pathology justifies the fixation of movable pancreas. 11. If from any cause the duct between the pancreas and the duodenum becomes closed, a new passage may be made for the pancreatic juice, or a pancreatic fistula may be established. 12. Stitches through the pancreatic parenchyma do no harm, and are tolerated, as in the kidneys, liver, and spleen. 13. In suturing the pancreatic duct the stitches should not pass into the interior of the duct, as concretions are liable to form on them. 14. Regeneration of the pancreas has been observed. 15. After complete extirpation of the pancreas great development of the glands of Galeati have been observed, and especially a karyokinetic increase in the epithelia, leading to the supposition that the missing gland might be thereby replaced. 16. The thermo-cautery or galvano-cautery ought not to be used in extirpation of the pancreas.

[1] Med Rec., New York, July 21, 1900, p. 98. [2] Brit. Med. Jour, Oct. 13, 1900, p. 1108. [3] Brit. Med. Jour., July 14, 1900, Epitome, No. 24. [4] Lancet, Sept. 8, 1900, p. 738. [5] Ibidem, Sept 15, 1900, p. 810. [6] Ibidem, Oct. 6, 1900, p. 1014. [7] Med. Rec., Oct. 20, 1900, p. 610. [8] Glasgow Med. Jour., March 1900, p. 190. [9] Annals of Surgery, June 1900, p. 706. [10] Lancet, July 28 and Aug. 11, 1900; and Brit. Med. Jour., Aug. 18, 1900, Epitome, No. 99. [11] Lancet, Aug 11, 1900, p. 428.

